# Cytotoxic Potential of Bioactive Compounds from *Aspergillus flavus*, an Endophytic Fungus Isolated from *Cynodon dactylon,* against Breast Cancer: Experimental and Computational Approach

**DOI:** 10.3390/molecules27248814

**Published:** 2022-12-12

**Authors:** Arjun Kumar Kalimuthu, Parasuraman Pavadai, Theivendren Panneerselvam, Ewa Babkiewicz, Joanna Pijanowska, Piotr Mrówka, Gopalan Rajagopal, Venkataraman Deepak, Krishnan Sundar, Piotr Maszczyk, Selvaraj Kunjiappan

**Affiliations:** 1 Department of Biotechnology, Kalasalingam Academy of Research and Education, Krishnankoil 626126, India; 2 Department of Pharmaceutical Chemistry, Faculty of Pharmacy, M.S. Ramaiah University of Applied Sciences, Bengaluru 560054, India; 3 Department of Pharmaceutical Chemistry, Swamy Vivekanandha College of Pharmacy, Elayampalayam 637205, India; 4 Department of Hydrobiology, Faculty of Biology, University of Warsaw, 101 Żwirki i Wigury Street, 02-089 Warsaw, Poland; 5 Department of Biophysics, Physiology and Pathophysiology, Medical University of Warsaw, 5 Chalubinskiego Street, 02-004 Warsaw, Poland; 6 Department of Experimental Hematology, Institute of Hematology and Transfusion Medicine, 5 Chocimska Street, 00-791 Warsaw, Poland; 7 Postgraduate and Research Department of Zoology, Ayya Nadar Janaki Ammal College, Sivakasi 626123, India; 8 Maternal and Fetal Health Research Centre, 5th Floor St. Mary’s Hospital, University of Manchester, Oxford Road, Manchester M13 9WL, UK

**Keywords:** *Cynodon dactylon*, *Aspergillus flavus*, endophytic fungus, secondary metabolites, anticancer, breast cancer, Bcl-2

## Abstract

Endophytic fungi are a diverse group of microorganisms that colonize the inter- or intracellular spaces of plants and exhibit mutual benefits. Their interactions with the host plant and other microbiomes are multidimensional and play a crucial role in the production of secondary metabolites. We screened bioactive compounds present in the extracts of *Aspergillus flavus*, an endophytic fungus isolated from the roots of the medicinal grass *Cynodon dactylon*, for its anticancer potential. An in vitro analysis of the Ethyl acetate extract from *A. flavus* showed significant cytostatic effects (IC_50_: 16.25 μg/mL) against breast cancer cells (MCF-7). A morphological analysis of the cells and a flow cytometry of the cells with annexin V/Propidium Iodide suggested that the extract induced apoptosis in the MCF-7 cells. The extract of *A. flavus* increased reactive oxygen species (ROS) generation and caused a loss of mitochondrial membrane potential in MCF-7 cells. To identify the metabolites that might be responsible for the anticancer effect, the extract was subjected to a gas chromatography-mass spectrometry (GC-MS) analysis. Interestingly, nine phytochemicals that induced cytotoxicity in the breast cancer cell line were found in the extract. The in silico molecular docking and molecular dynamics simulation studies revealed that two compounds, 2,4,7-trinitrofluorenone and 3α, 5 α-cyclo-ergosta-7,9(11), 22t-triene-6beta-ol exhibited significant binding affinities (−9.20, and −9.50 Kcal/mol, respectively) against Bcl-2, along with binding stability and intermolecular interactions of its ligand-Bcl-2 complexes. Overall, the study found that the endophytic *A. flavus* from *C. dactylon* contains plant-like bioactive compounds that have a promising effect in breast cancer.

## 1. Introduction

Cancer is one of the most devastating diseases worldwide, responsible for approximately 10 million deaths in 2020, or about one in every six deaths. It has been estimated that the number of cancer-related deaths may increase to 13.1 million by 2030 [1]. Each cancer case is different and unique. The origin of the tissue, the types of genetic mutations, and the surface protein markers are used to systematically analyze and classify cancers for better prevention, diagnostics, and treatment [2]. Of the various types of cancer, breast cancer is one of the most frequent among women, with an estimated 1.38 million new cases every year [3]. It ranks second in the world based on its incidence and mortality rates [4]. Although the exact mechanism that triggers cancer transformation has not been fully established, several genetic mutations and epigenetic dysregulations in genes related to proliferation, cell differentiation, and death are frequently observed in cancer cells [5]. In healthy breast epithelial cells, the pro-apoptotic and anti-apoptotic signals are closely controlled. The members of the B-cell lymphoma-2 (Bcl-2) family of proteins, composed of pro-apoptotic and anti-apoptotic protein, play a pivotal role in the mitochondria-mediated apoptosis. Dysregulation of this balance results in breast tumorigenesis followed by acquired resistance to treatments, including molecularly targeted therapies, radiation, and chemotherapies [6].

The last couple of decades have brought about great improvements in the treatment of breast cancer patients. In many cases, if diagnosed early, breast cancer is considered curable. The current treatment strategies include surgery and radio- and chemotherapy. Targeted therapy using small drug inhibitors and immunotherapy is now being studied for different disorders, including cancer. The treatment regime includes classic chemotherapeutic medicines, such as taxol (Paclitaxel), doxorubicin (Adriamycin), or vinblastine (Velbe); small-molecule targeted drugs like lapatinib, neratinib, or imatinib mesylate (Gleevec/Glivec), a tyrosine kinase inhibitor; and monoclonal antibodies, particularly trastuzumab (Herceptin) [7]. Despite these sophisticated treatments for breast cancer, in some cases the disease develops a resistance to therapy. Another aspect to consider is the influence of treatment on a patient’s life, which is significantly affected by the adverse side effects of the therapy [8]. A couple of the strategies being used to address the disadvantages of breast cancer treatments are the optimization of treatment protocols (multiple therapies, algorithms of procedures, doses, etc.) and the search for new, effective compounds that could selectively inhibit cancer cells’ survival pathways, minimizing the side effects. Therefore, it is vital to seek novel effective therapeutic agents with limited side effects for treating breast cancer. The overexpression of Bcl-2 in breast cancer cells is involved in the cells’ proliferation and survival and has been credited with their ability to developing a resistance to therapy through the evasion of apoptosis [9].

Natural compounds derived from plants, bacteria, fungi, and algae, which may either be from terrestrial or marine environments, play a significant role in the development of cancer therapeutics [10,11,12]. Fungi are a diverse kingdom of living beings estimated to include 2.2 to 3.8 million species, and the about 148,000 already described natural compounds derived from them could be a potent source of therapeutic drugs [13]. Unfortunately, only around five percent of these fungi have been cultivated in the laboratory, and thus only a small fraction of fungal-derived compounds have being studied so far [14,15]. The huge variety of metabolites produced by fungi includes different structural types of compounds: amino acids, aromatic chemicals, anthracenones, butenolides, cytochalasans, macrolides, naphthalenones, terpenes, and pyrones [16,17,18]. Some factors that make fungi an attractive source of medicinal compounds include their rapid growth, culture conditions, high cell density, and ease of genetic modification [19]. Currently, more and more research is being focused on fungal metabolites and is revealing their promising anticancer properties [20].

Endophytic fungi (phyla: fungi) live inter- or intracellularly in various plant species and produce a plethora of substances that aid in the growth and survival of plants [21]. They are a promising source of secondary metabolites, which include enzymes [22], antimicrobials [23], and anticancer chemicals, such as bikaverine [20] and triterpenes [24]. Many unique natural compounds with antioxidant and anticancer properties remain to be discovered from them.

*Cynodon dactylon*, often known as “Durva Grass”, “Bermuda grass”, “Dog’s Tooth grass”, “Bahama grass”, “Devil’s grass”, “Couch grass”, “Indian Doab”, “Scutch grass”, “Dhub”, and “Arugampul”, is a plant of immense religious significance that is reportedly used in the Ayurvedic, Siddha, Unani, Nepalese, and Chinese medical systems [25]. *C. dactylon* extract has been successfully tested in preclinical studies for its antitumor activity. For example, in vivo studies of *C. dactylon* root extract in diethyl nitrosamine (DEN) induced liver cancer in mice have shown its significant anticancer activity [26]. As we know that *C. dactylon* is home endophytic fungi that can be a source of unique substances, the present study is aimed at investigating the potential antitumor properties of the bioactive compounds from endophytic fungi in the in vitro breast cancer model. 

## 2. Materials and Methods

### 2.1. Materials and Reagents

Dulbecco’s Modified Eagles medium (DMEM), rhodamine-123, fetal bovine serum (FBS), tris-EDTA buffer, propidium iodide (PI), ethidium bromide (EtBr), annexin-V, 4′,6′-diamino-2-phenylindole, fluorescein isothiocyanate (FITC), amphotericin B, phosphate buffered saline (PBS), 2′,7′-dichlorofluorescein diacetate (H_2_DCF-DA), penicillin G sodium, streptomycin sulphate, and lactophenol were purchased from HiMedia Laboratories, Mumbai, India. The other organic solvents and fine chemicals were of analytical grade and were purchased from Merck and SD Fine Chemicals, Mumbai, India.

### 2.2. Cell Lines and Culture Media

The MCF-7 cell line (human breast cancer epithelial cells) was obtained from the National Centre for Cell Science, Pune, India. For the culture maintenance and in vitro cytotoxicity experiment, MCF-7 cells were cultured in a culture flask or on plates (Falcon, Sigma-Aldrich, Bengaluru, India) in DMEM, supplemented with 20% heat inactivated FBS, 1% penicillin, streptomycin, and amphotericin B in stable conditions of 37 °C and 5% CO_2_ in a humidified atmosphere. A passage no higher than 20 was used (P18 in most of the experiments). The cells were frequently tested against mycoplasma according to the laboratory protocol [27].

### 2.3. Collection of Plant and Isolation of Endophytic Fungus

Nourishing free growth *C. dactylon* plants were gathered from Sundarapandiam village, Virudhunagar Dist., Tamil Nadu, India (latitude 9.59721° N, longitude 77.67405° E). The location is characterized by a high level of vegetation development and a large variety of plants, including several species of grass. Immediately after collecting the grass, the samples were transported to the laboratory and carefully cleansed with distilled water. On the collection day, fungal endophytes were isolated as described by Tapfuma et al. [28]. The *C. dactylon* plant root was chopped into tiny (1 cm) pieces under sterile conditions. These were washed using running tap water for 2–5 min to remove dirt particles and undesired debris, followed by rinsing with deionized water. The chopped plant roots were then sterilized by soaking in 70% ethanol for 1 min, followed by 1% sodium hypochlorite for 6 min. The root sample was then cleaned again for 1 min in 70% ethanol before being rinsed with sterile deionized water. The sterilized root sample was coarsely crushed and serially diluted in sterile water using a sterile mortar and pestle. Dilutions ranging from 10^−2^ to 10^−6^ were plated on potato dextrose agar (PDA, pH = 5.6) and incubated at 27 °C for 4–7 days. The plates were examined after 48 h for the formation of fungal colonies. The morphology, texture, and color of the colonies were recorded. Lactophenol cotton blue (LCB) wet mount was used to observe the microscopic morphology of the fungi.

### 2.4. Molecular Identification of Endophytic Fungi 

The endophytic fungus isolated from *C. dactylon* was identified using molecular gene sequencing according to the methods used by Sette et al. 2006 [29]. The 200 mg of fungal mycelial mat were mashed with a pestle and mortar in 500 µL of extraction buffer (200-mM Tris–HCl (pH = 8.0), 25-mM EDTA (pH = 8.0), 250-M NaCl, 10% CTAB) according to the Cetyltrimethyl ammonium bromide (CTAB)-phenol–chloroform–isoamyl alcohol technique (CTABPCI). The mashed fungal mycelia was then transferred to a new tube, where 3 *µ*L proteinase K and 3 *µ*L RNase were added, vortexed, and incubated at 37 °C for 1 h. After incubation, the tubes were placed in a 65 °C water bath for 10 min. After adding one volume of phenol, chloroform, and isoamyl alcohol (25:24:1), the solution was well mixed for 5 min before being centrifuged at 12,000 rpm for 5 min. The clear aqueous phase was collected and combined with one volume of chloroform: isoamyl alcohol (24:1) mixture before centrifugation at 12,000 rpm for 5 min to recover the aqueous phase. One volume of ice-cold isopropanol was added and the mixture was held overnight at −20 °C for DNA precipitation. The DNA was precipitated with 100% ethanol after being centrifuged at 10,000 rpm for 5 min and was then washed twice with 1 mL of 70% ethanol before being resuspended in 200 *µ*L of deionized water or 1× TE (200 mM Tris–HCl (pH = 8.0), 20 mM EDTA (pH = 8.0)) buffer. A portion of the eluted fungal DNA was electrophoresed on a 0.8% agarose-EtBr gel, and the concentration was calculated using Qubit 3.0. 

PCR with universal primers recognizing the internal transcribed spacer (ITS) region of the fungal DNA, ITS 5 (TCCTCCGCTTATTGATATGC) forward and ITS 4 (GGAAGTAAAAGTCGTAACAAGG) reverse, was used to identify the species of isolated fungi. Amplified DNA fragments were validated and purified with a GeneJET PCR purification kit (Thermo Scientific, EU-Lithuania, Vilnius, Lithuania) to exclude primer dimer and other carryover contaminants. Using a 2% agarose gel and a 100-bp DNA ladder as a size reference, the product’s quality was evaluated and determined to be acceptable for sequencing. The Big Dye^®^ Terminator 3.1 sequence kit was used to purify and prepare the PCR-amplified products for sequencing (Applied Biosystems, Foster City, CA, USA). According to the manufacturer’s instructions, the denatured products were sequenced in both the forward and reverse directions using a Genetic Analyzer 3500 (Life Technologies Corporation, Applied Biosystems^®^, Carlsbad, CA, USA). To validate the species, the sequences were aligned and modified using Mega software version 10. The annotated ITS rDNA contig was submitted to GenBank and assigned an entry number.

### 2.5. Evaluation of Anticancer Activity

#### 2.5.1. MTT Assay

The cytotoxic properties of the endophytic fungal extract were assessed using metabolic MTT assay in MCF-7 cells. Briefly, MCF-7 cells were seeded into 96-well microtiter plates and cultured at 37 °C with 5% CO_2_ for 24 h to reach confluency. The cells were further treated with various concentrations of the endophytic fungal extract (100, 50, 25, 12.5, 6.25, 3.125 µg/mL). After 48 h, MTT assay was performed according to the standard procedure [30]. Untreated cells served as control. The absorbance was measured at 570 nm using a microplate reader (Bio-Rad, Hercules, CA, USA). The percentage of viability was calculated using the following formula:

Cell viability (%)* = (A_(mean absorbance of extract-treated cells)_/A_(control cells)_) × 100; *n = 3

where n is the number of independent experiments.

#### 2.5.2. Detection of Cytotoxicity Using Dual Staining Assay

The MCF-7 cells were cultured in 24-well microtiter plates and treated with the IC_50_ concentration of the endophytic fungal extract for 48 h. After 48 h of treatment, the cells were rinsed in ice-cold 1× PBS (phosphate buffered saline) and then stained with two fluorescent DNA binding stains [(10 μg/mL) (Acridine orange and Ethidium bromide)] [31]. After 30 min of incubation, the cells were observed for apoptosis under a fluorescent microscope (Labomed TCM 400 Inverted Binocular Microscope, USA, using Image aR Pro software) at 40× magnification. The untreated cells served as a control for the study. The percentage of the apoptotic cells was calculated using the following formula: (Total number of apoptotic cells/Total number of normal cells) × 100.

#### 2.5.3. Apoptosis Analysis Using Flow Cytometry

MCF-7 cells were seeded on 24-well microtiter plates and cultivated overnight at 37 °C in a CO_2_ incubator. The cells were then treated with the IC_50_ concentration of the fungal extract for 48 h. Following incubation, the cells were washed in PBS and centrifuged for 5 min at 500× *g* in 4 °C. The supernatant was then removed, and the cell pellets combined to a concentration of 1 × 10^5^ mL^−1^ in an ice-cold 1× binding buffer. Next, 1 μL of annexin V-fluorescein isothiocyanate reagent and 1 μL of propidium iodide (PI) were added to the tubes on ice. The tubes were then kept on ice and incubated in the dark for 15 min, and 400 μL of ice-cold 1× binding buffer were meticulously mixed in. Within 30 min, a flow cytometry analysis was performed (BD FACS calibur-Becton Dickinson, CytExpert v 1.2.11.0) [32].

#### 2.5.4. Assessment of Mitochondrial Transmembrane Potential

The mitochondrial transmembrane potential was determined by using a lipophilic dye, Rhodamine-123 [33]. MCF-7 cells were seeded in the 24-well plate and maintained for 24 h in 5% CO_2_, 37 °C conditions to reach the exponential growth phase. Then, the cells were exposed to IC_50_ of the fungal extract. After 48 h of incubation, the cells were stained with Rhodamine-123 dye for 30 min. Subsequently, they were washed with PBS and fixed with paraformaldehyde (4%) for 30 min. The morphological alterations and the permeability of the membrane were examined under the fluorescent microscope. The untreated cells served as a control.

#### 2.5.5. Nuclear Integrity Measurement

MCF-7 cells were treated with the endophytic fungal extract for 48 h in a 24-well flat bottom microplate as described in the previous sections. Then, the cells were washed with PBS and fixed in paraformaldehyde (4%) for 30 min. After this, the cells were washed in Triton X100 (0.4%) for 20 min and washed in PBS. Next, the cells were stained with DAPI (0.5 µg mL^−1^) in the dark for 1 min and washed with PBS. A fluorescent microscope with an appropriate filter was used to capture images of the cells [30].

#### 2.5.6. Determination of Reactive Oxygen Species (ROS)

The level of intracellular ROS was measured using 2′,7′-dichlorofluorescein diacetate. The MCF-7 cells were cultured for 24 and 48 h after being exposed to 10% FBS and the IC_50_ concentration of the endophytic fungal extract. The cells were then washed twice with PBS before being tagged with 10 M H_2_DCF-DA according to the manufacturer’s protocol [32]. The fluorescence intensity was measured using a fluorescence spectrophotometer (SpectramaxM2 fluorescence spectrophotometer, Molecular devices, San Jose, CA, USA) at 475 nm (λ_ex_) and 525 nm (λ_em_).

### 2.6. Gas Chromatography-Mass Spectrum (GC-MS) Analysis

We used sonication to lyse and homogenize fungal mycelia for the extraction of secondary compounds [34]. Around 500 mg of fungi (pure mycelial culture) were taken and dispersed in 10 mL of an 80% ethyl acetate solution. The contents were subjected for sonication in the ultrasonic bath sonicator (Elma Ultrasonic Cleaner S100H, Mumbai, India) at a 80 W cm^2^ intensity for 0.5 pulse cycles at a temperature of 40 °C. After the process of sonication, the contents were filtered using a Whatman No. 1 paper filter to eliminate the mycelia, and the remaining filter was centrifuged at 2500 rpm. The liquid containing the secondary metabolic compounds of the endophytic fungi was determined by GC-MS analysis. A Shimadzu Make QP-2010 on a non-polar 60 M RTX 5MS column was used for the GC-MS analysis, with helium as the carrier gas, containing 15 psi as constant pressure.

### 2.7. In Silico Molecular Docking

The GC-MS spectrum provided a list of biologically active phytochemicals contained in the isolated endophytic fungal metabolic extract. The compounds were utilized for molecular docking studies against the overexpressed anti-apoptotic protein receptor (Bcl-2, PDB id: 6O0K, Resolution: 1.62 Å) of breast cancer. The three-dimensional molecular structure of compounds (ligands) from the endophytic fungal extract was created using Chemsketch software. The Chemistry at Harvard Molecular Mechanics (CHARMm) force field was used to optimize the ligands and minimize energy. The generated ligand structures were converted into the “.pdb” file format using BIOVIA|Discovery Studio Visualizer v20.1.0.19295 software (Accelrys Software Inc., San Diego, CA, USA). The target protein model was retrieved from the RCSB’s protein data repository [35].

### 2.8. Molecular Dynamics (MD) Simulation

MD simulation studies were performed to investigate the binding stability of the top scored compounds of the endophytic fungal extract with the anti-apoptotic protein receptor (Bcl-2) of breast cancer [36]. Using the Desmond dynamic package 2017 in Schrodinger (Academic version) in a Linux environment, the time-dependent change of the complexes was calculated over 200 ns [36]. The complex of the identified phytocompounds with the Bcl-2 receptor was created using the OPLS (optimal potentials for liquid simulations)-2005 force field [37]. Additionally, a water model was created using the established SPC water model at distances of 10 Å units from an orthorhombic periodic boundary [38]. Further, the electric charges were neutralized by adding the required number of counter ions, and, before the MD simulation process started, the system decreased its energies through heating and equilibrium processes. The Nose-Hoover approach with the NPT (isothermal-isobaric ensemble) was used to apply a 300 K temperature and one atmospheric pressure (1.01325 bar) throughout the system’s final manufacturing stage, which lasted up to 200 ns [39,40]. The best confirmations were chosen with regard to the complex’s interactions and dynamical characteristics.

### 2.9. Statistical Analysis

SPSS Statistics version 20.0 was used for all statistical analyses. A one-way ANOVA followed by Dunnett’s multiple comparisons post hoc test was used, with the significance level set at *p* < 0.05 or lower. Representative results of at least three independent repeats are shown in each experiment [41].

## 3. Results

### 3.1. Isolation of Fungi and Microscopic Examination

**Figure 1:**
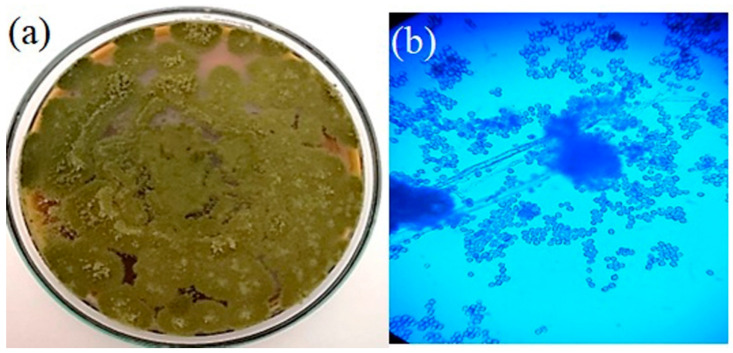
Morphology of the colonies of the endophytic fungus: (**a**) image of the colonies in Potato Dextrose Agar; (**b**) microscopic view of the fungal spores (LCB).

*C. dactylon* roots served as the source of the fungi. Four endophytic fungi were selected for the study based on the morphology of the colonies and the antioxidant potentials of their extracts. In potato dextrose agar, the fungi colony looks powdery and has an olive-green conidial appearance. Later, the chosen fungus was sub-cultured and purified for further analysis. Figure 1a depicts the colony morphology of the endophytic fungi, and Figure 1b illustrates the microscopic view of the mycelia and the fungal spores using the lactophenol cotton blue mounting (LCB) technique.

### 3.2. Phylogenetic Analysis

**Figure 2:**
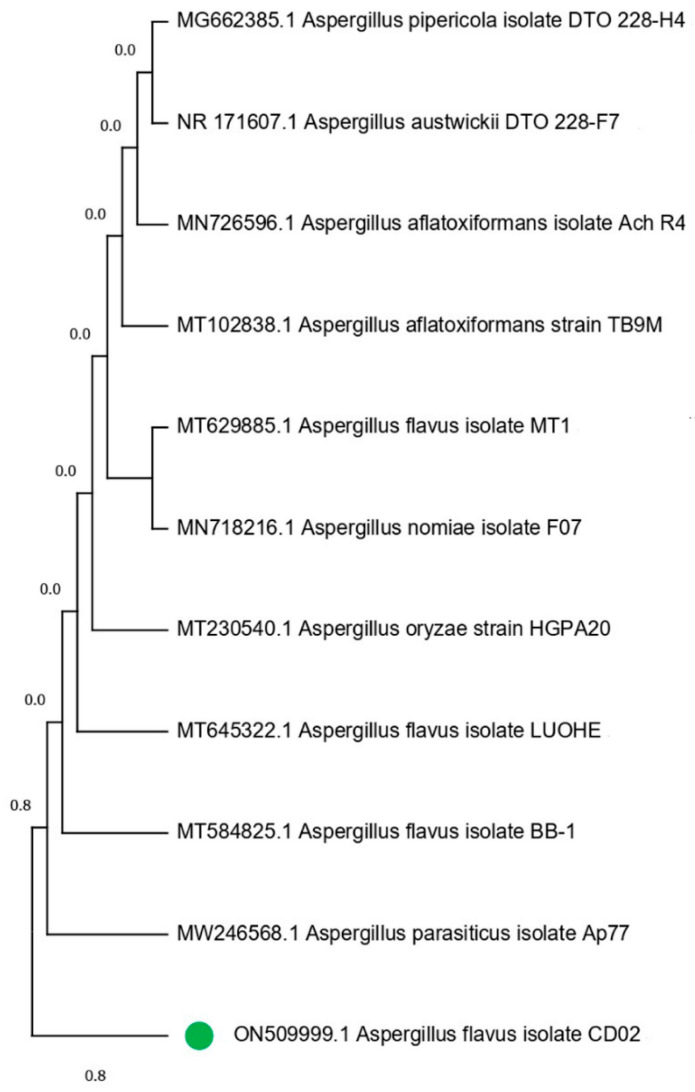
*Aspergillus* phylogeny tree based on analysis of ITS sequence. The isolate CD02 (with bootstrap index > 50%) is marked with green dot.

Through the systematic selection of isolates from the fungal culture, all of the colonies were categorized according to their morphological parameters, such as the shape and color of the colony, and their reverse media color. Each fungal colony was purified by transplanting sequential hyphal ends over 2–3 passes. One of the four isolates, CD02, showed higher antioxidant activity and was chosen for further investigation. The selected fungal isolate was identified using PCR with ITS4 and ITS5 as forward and reverse primers, respectively. The BLASTn analysis revealed that the strain CD02 belongs to the genus *Aspergillus*, and the species was identified as *Aspergillus flavus* (Genbank No: ON509999.1). The rRNA sequence of the isolate, CD02, has a 96% identity with *Aspergillus flavus*. The 18S rRNA sequence of these fungal isolates was aligned with reference strains from GenBank, and this formation was used to build a phylogenetic tree (Figure 2) showing the relations of the newly identified fungal strain with other members of the genus *Aspergillus*. The neighbor joining tree demonstrated a tight link between the strains recovered in this investigation and *Aspergillus flavus* MT584825.

### 3.3. Anticancer Activity

#### 3.3.1. Cytotoxicity Assay

**Figure 3:**
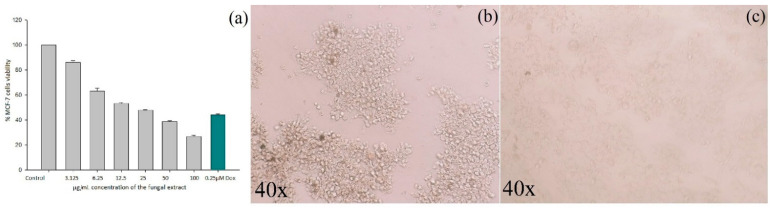
Cytotoxic effects of 48 h treatment with serial dilutions of *A. flavus* extract on MCF-7 cells. (**a**) The percentage loss of viability of cells determined with MTT assay is shown. Values are mean ± standard deviation of triplicate measurements (*p* < 0.05). (**b**) Morphology of the control cells; (**c**) morphology of cells treated with 16.25 μg/mL.

The cytotoxic efficacy of the *A. flavus* extract against MCF-7 cells was assessed using metabolic MTT assay, a viability test directly measuring the cellular redox potential in a population of cells. The IC_50_ value of the fungal extract was found to be at 16.25 μg/mL. Figure 3a illustrates the percentage of the viability of the cells at various concentrations (μg/mL), and Figure 3b,c shows the untreated control cells (Figure 3b) and changes in morphology of the MCF-7 cells after being treated with *A. flavus* extract (Figure 3c). The IC_50_ concentration of the *A. flavus* extract (16.25 μg/mL) was then selected for further analysis. Doxorubicin (0.25 μM) served as a positive control for this experiment [42]. The statistical data are presented in Supplementary Table S1.

#### 3.3.2. Analysis of Apoptosis Using Dual Staining

**Figure 4:**
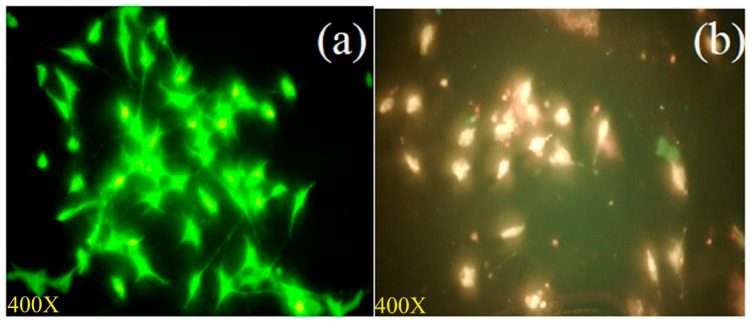
Fluorescent microscopy images of apoptotic structural changes of Acridine orange/Ethidium bromide (AO/EtBr) stained MCF-7 cells upon *A. flavus* extract exposure: (**a**) Untreated control cells; (**b**) cells treated with 16.25 μg/mL of *A. flavus* extract for 48 h.

MCF-7 cells treated with the IC_50_ concentration of the *A. flavus* extract were stained with AO/EtBr to visualize live and dead cells. Using fluorescent microscopy, cells were observed to stain bright orange/red, depicting the dead cell form, after treatment with the *A. flavus* extract, whereas cells stained green in the untreated control, depicting the live form (Figure 4). 

#### 3.3.3. Assessment of Apoptosis by Flow Cytometer

**Figure 5:**
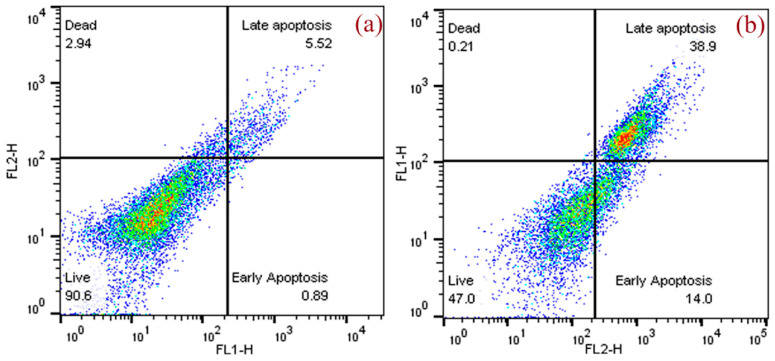
Flow cytometry Annexin V/Propidium Iodide (PI) analysis shows increased apoptosis of MCF-7 cells treated with *A. flavus* extract: (**a**) untreated control; (**b**) MCF-7 cells treated with *A. flavus* extract.

Apoptosis was also studied using annexin V and PI staining. Figure 5 shows the results of the flow cytometry analysis of the *A. flavus* extract in MCF-7 cells treated with 16.25 μg/mL of extract for 48 h. Early apoptosis was observed in 14% of treated cells, which was significantly higher than in the control cells (0.89%). The percentage of cells in late apoptosis induced by *A. flavus* extract was also significantly higher (38.9%) compared to the control cells (5.52%). Table 1 displays the percentages of live, early apoptosis, late apoptosis, and dead cells.

#### 3.3.4. Measurement of Mitochondrial Transmembrane Potential

**Figure 6:**
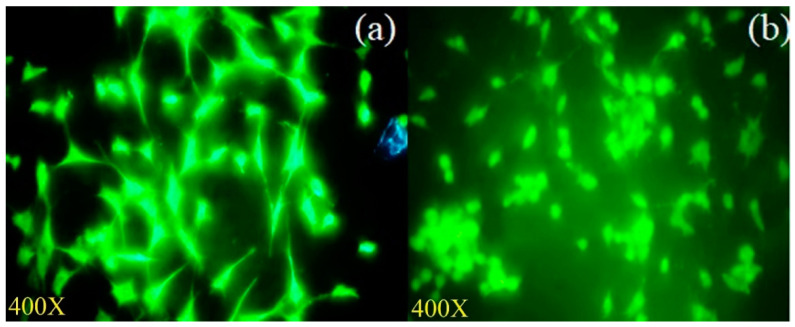
Fungal extract reduces the mitochondrial transmembrane potential as seen under fluorescent microscope using rhodamine-123 staining: (**a**) Untreated control cells; (**b**) depolarized mitochondrial membrane in MCF-7 cells treated with IC_50_ of *A. flavus* extract.

The changes in mitochondrial membrane potential were examined using rhodamine-123 dye in MCF-7 cells with and without treatment with the *A. flavus* extract for 48 h (IC_50_). After 24 h, the mitochondrial membrane potential in the MCF-7 cells treated with the *A. flavus* extract was significantly reduced (Figure 6a,b). Some morphological changes in the *A. flavus* extract-treated cells and their mitochondria can also be observed.

#### 3.3.5. Assessment of Nuclear Integrity by DAPI Staining

**Figure 7:**
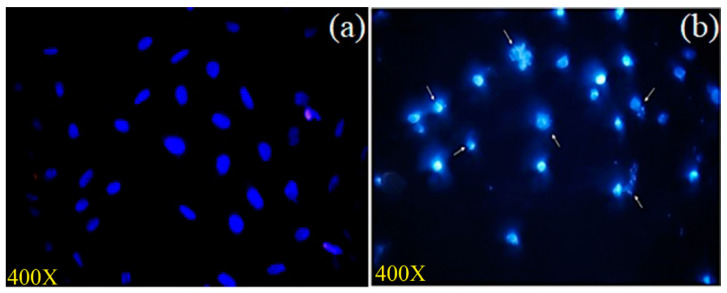
MCF-7 cells stained with DAPI: (**a**) Untreated control; (**b**) MCF-7 cell treated with 16.25 μg/mL of extract chromatin. Condensation and fragmentation (indicated by arrow), nucleus shrinkage, and blebbing were also observed.

As the *A. flavus* extract was found to induce apoptosis in MCF-7 cells, the morphological changes of the cellular nuclei were analyzed using DAPI staining (Figure 7). DAPI stain binds to the adenine and thymine regions of the nuclear DNA and produces a blue fluorescence. After a 48 h treatment of the cells with *A. flavus* extract, we observed shrinkage of the cells, condensation of the chromatin, and damage to the nuclear DNA (Figure 7). In addition, compared to the control, the *A. flavus* extract-treated MCF-7 cells showed a decreased intensity of blue fluorescence. All of these phenomena confirm the apoptotic nature of the cell death induced by the *A. flavus* extract [43].

#### 3.3.6. Assessment of Intracellular Reactive Oxygen Species

**Figure 8:**
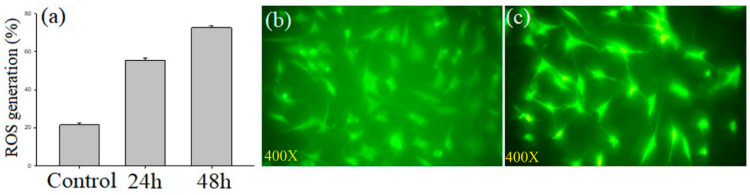
Effects of 16.25 μg/mL of *A. flavus* extract on ROS generation (percentage of control) in MCF-7 cancer cells. Results expressed as mean ± standard deviation of triplicate measurements (*p* < 0.05) (**a**); ROS generation measured as relative fluorescence intensity using fluorescence microscope. (**b**) Image of untreated control cells; (**c**) increased ROS in *A. flavus* extract-treated cells.

An important factor in the development and biology of cancer is the imbalance between the levels of reactive oxygen species and antioxidants. Figure 8a displays the results of the induction of ROS by the *A. flavus* extract in MCF-7 cells, using the H_2_DCF-DA staining method. Increased green fluorescence was seen in the cells treated with 16.25 μg/mL of the *A. flavus* extract as compared to the control (Figure 8b,c), indicating the onset of apoptosis in the MCF-7 cells. The statistical data are presented in Table S2.

### 3.4. GC-MS Analysis

**Figure 9:**
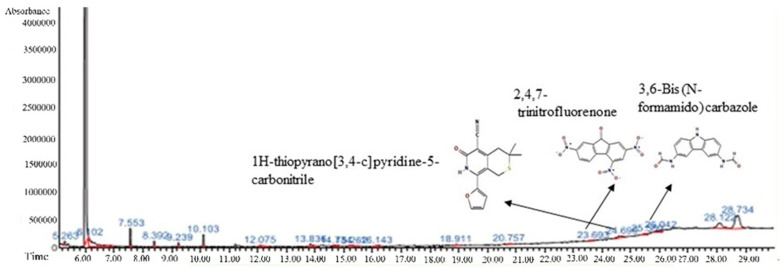
Phytochemical profiling of *A. flavus* extract.

The GC-MS spectra of the *A. flavus* extract revealed the presence of bioactive phytochemicals with corresponding peaks at the time of retention. In total, nine bioactive compounds were identified from the GC-MS spectrum (Figure 9). The major bioactive phytochemicals present were tritetracontane, heptadecanoic acid, methyl 2,8-dimethyltridecanoate, 2,3,4-trimethyllevoglucosan, 2,4,7-trinitrofluorenone, 1H-thiopyrano [3,4-c] pyridine-5-carbonitrile, 3,6-Bis (N-formamido) carbazole, 3α, 5α,-cyclo-ergosta-7,9(11),22t-triene-6β-ol, and 1H-isoindole-1, 3 (2H)-dione. Table 2 displays the identified phytochemicals and their structures.

### 3.5. Molecular Docking (MD)

**Figure 10:**
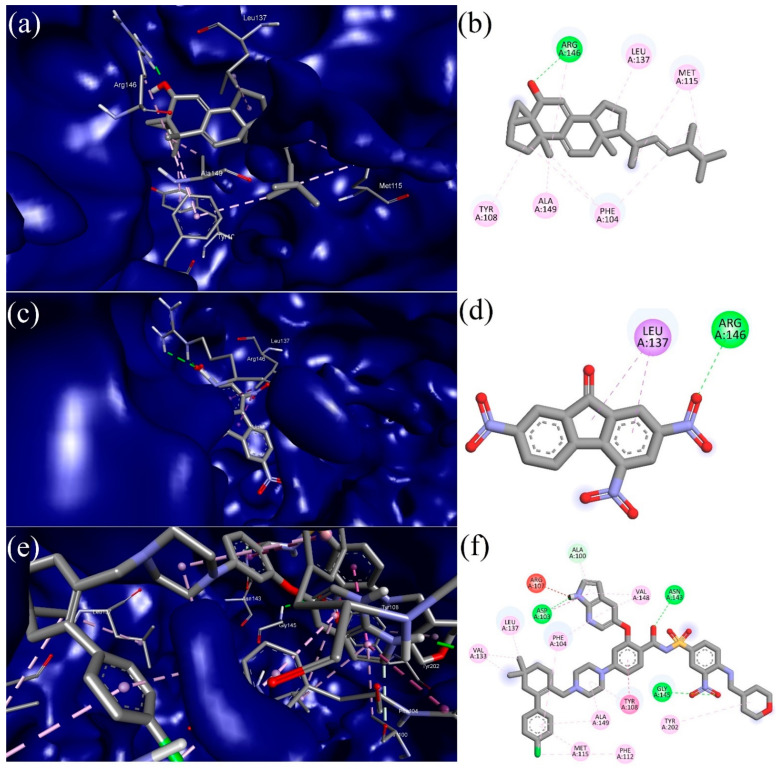
Images represent 3D (left) and 2D (right) maps of protein–ligand complex interaction between: (**a**,**b**) 3α, 5α-cyclo-ergosta-7,9(11),22t-triene-6β-ol and Bcl-2 receptor; (**c**,**d**) 2,4,7-trinitrofluorenone and Bcl-2 receptor; and (**e**,**f**) Venetoclax and Bcl-2 receptor.

Molecular docking (MD) studies were used to examine the intermolecular interaction between the target protein (Bcl-2) and the bioactive phytochemicals. The identified bioactive phytochemicals showed a strong intermolecular interaction and significant binding affinities against Bcl-2. According to molecular docking studies, the binding energies of the bioactive phytochemicals ranged from −4.50 to −9.50 Kcal/mol, as shown in Table 3. Two bioactive compounds, 2,4,7-trinitrofluorenone (−9.20 Kcal/mol) and 3α, 5α-cyclo-ergosta-7,9(11),22t-triene-6β-ol (−9.50 Kcal/mol), demonstrated better binding affinities against the anti-apoptotic protein receptor (Bcl-2) of breast cancer as compared to the standard drug Venetoclax (−10.90 Kcal/mol). Two top scoring compounds, along with Venetoclax as a reference drug, were taken for further MD simulation studies to confirm the stability of the complexes. Furthermore, the protein–ligand interaction profiler online tool (https://plip-tool.biotec.tu-dresden.de/plip-web/plip/index, accessed on 21 November 2022) was used to visualize the intermolecular interactions between the ligands and the target protein (Bcl-2). The visualized result indicates that the chosen compound, 2,4,7-trinitrofluorenone, showed a docking score (−9.20 Kcal/mol) against the anti-apoptotic protein Bcl-2, and it formed three hydrophobic interactions (PHE104A (3.28 Å), ASP111A (3.93 Å), LEU137A (3.55 Å)) with the receptor, as depicted in Figure 10a,b. The top scored bioactive phytocompound, 3α, 5α-cyclo-ergosta-7,9(11),22t-triene-6β-ol, established contact with Bcl-2 through seven hydrophobic bonds (PHE104A (3.41 Å), TYR108A (3.82 Å), TYR108A (3.97 Å), LEU137A (3.57 Å), ARG146A (3.91 Å), PHE153A (3.76 Å), VAL156A (3.69 Å)), and two hydrogen bonds (ARG156A (2.20 Å), ARG156A (1.98 Å)), as presented in Figure 10c,d. The standard drug Venetoclax formed eleven hydrophobic bonds (PHE104A (3.17 Å), PHE104A (3.68 Å), PHE104A (3.99 Å), PHE104A (3.84 Å), ASP111A (3.76 Å), VAL133A (3.96 Å), GLU136A (3.74 Å), LEU137A (3.76 Å), VAL148A (3.75 Å), ALA149A (3.99 Å), TYR202A (3.62 Å)), three hydrogen bonds (ASP103A (1.93 Å), TYR108A (3.25 Å), ASN143A (2.03 Å)), and one salt bridge (ASP111A (4.73 Å)) with the target anti-apoptotic protein Bcl-2, as presented in Figure 10e,f.

### 3.6. Molecular Dynamics Simulation

**Figure 11:**
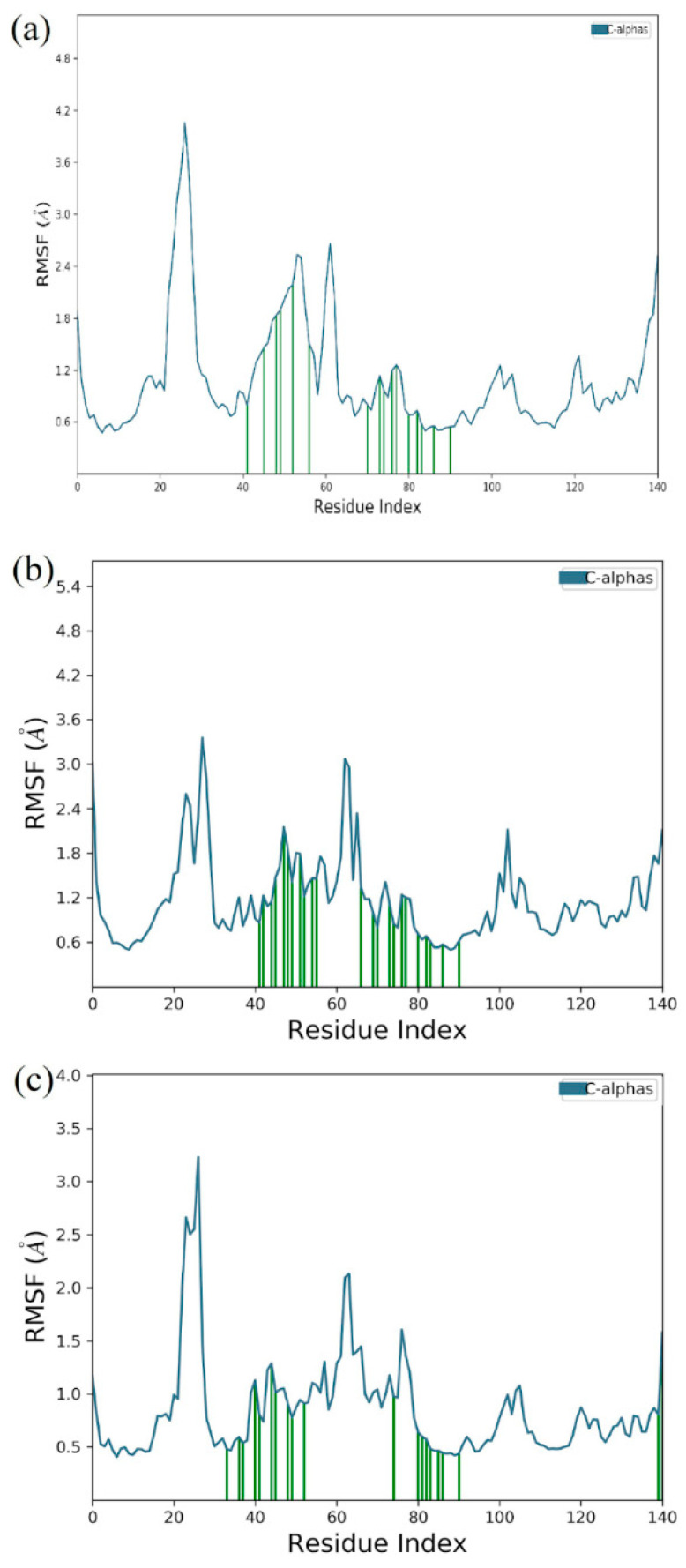
Root mean square fluctuation (RMSF) of 3α, 5α-cyclo-ergosta-7,9(11),22t-triene-6β-ol and Bcl-2 receptor complex (**a**); 2,4,7-trinitrofluorenone and Bcl-2 receptor complex (**b**); and Venetoclax and Bcl-2 receptor complex (**c**).

**Figure 12:**
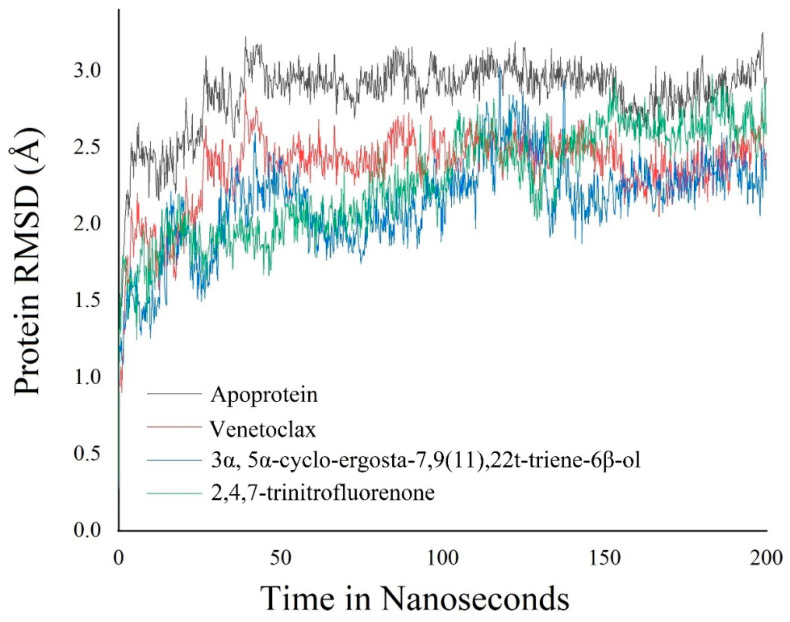
Root mean square deviation (RMSD) of backbone atoms of APO and its complex with Bcl-2 receptor; 2,4,7-trinitrofluorenone and complex with Bcl-2 receptor; and Venetoclax and complex with Bcl-2 receptor.

The molecular dynamic simulation was carried out for 3α,5α-cyclo-ergosta-7,9(11),22t-triene-6β-ol, 2,4,7-trinitrofluorenone, and standard Venetoclax complexed with Bcl-2 protein for 200 ns. The molecular dynamic simulation trajectory events of Apo protein Bcl-2 showed fluctuation to 3.2 Å and retained its stability to 3 Å throughout the simulation period, with an average RMSD of 2.851 Å. The phytoconstituent 3α, 5α-cyclo-ergosta-7,9(11),22t-triene-6β-ol, 2,4,7-trinitrofluorenone complex showed fluctuation initially up to 3 Å and retain its stability thereafter until the end, with 2.8 Å throughout the simulation period with an average RMSD of 2.14 Å, while the other selected phytoconstituent, 2,4,7-Trinitrofluorenone, showed 2.98 Å and retained its stability to 2.6 Å throughout the simulation period with an average of 2.278 Å. The Venetoclax complex showed the fluctuation of 2.8 Å and retained its stability to 2.4 Å throughout the simulation period, with an average RMSD of 2.368 Å (Figure 11). An RMSF analysis of all complexes shows no major fluctuation due the binding of amino acids with the key functional groups of ligands (Figure 12).

## 4. Discussion

Despite the progress being made in cancer treatment, new approaches are still needed to overcome tricky situations that can affect the current therapies, such as the development of drug resistance, relapse, secondary cancers, and adverse effects of the therapeutic procedures. A potent source of new compounds for cancer treatment can be found in the greatest chemical laboratory on earth—nature. Sustainable drug discovery programs to find new therapeutic phytocompounds with anticancer potentials are run in parallel to the chemical engineering of drug molecules [44]. In recent years, the use of phytomedicines for cancer treatment has noticeably increased [45]. Phytomedicines are already used for the successful treatment of many diseases and overcome many harmful adverse effects of the standard methods [46]. Plant derived medicines are also used more and more often in combination therapy with other medicines to reduce adverse effects and to increase the effectiveness of the treatment [47,48]. Almost 60% of the anticancer drugs currently being used for anticancer therapy are obtained from natural sources [49,50]. In this study, we found highly effective phytocompounds with anticancer potential that come from a fungus, *A. flavus*, which is found in medicinal plants.

Interestingly, in recent years, materials and products with considerable medicinal values have been found to be produced by the microorganisms associated with plants, rather than by the actual plants [51]. The endophytic bacteria and fungi that thrive in apparently healthy internal plant tissues are either facultative or obligate symbiotic microbes [52]. As mentioned earlier, the *Aspergillus* species are an important source of novel active phytochemicals with potential therapeutic values [53]. The biggest advantages of endophytic fungi are their fast renewal, relatively easy cultivation, cost-effectiveness, and environmental safety [54]. Numerous bioactive substances with a wide range of activities have been found in endophytic fungi over the last 20 years [55]. These bioactive compounds could be classified as flavonoids, alkaloids, terpenoids, diterpenes, sesquiterpenes, polyphenols, phenolic acids, indole derivatives, aliphatic compounds, pyridine, pyrazolidine, pyrimidine, thiazole derivatives, etc. In this study, the bioactive compounds present in the endophytic fungi *A. flavus*, isolated from the root of *C. dactylon*, have been determined to have anticancer properties against human breast cancer cells. *C. dactylon* (Family: Poaceae) was described as containing numerous bioactive compounds, such as vitexin, orientin, homoorientin, friedlein, beta-carotene, triterpenoids, ergonovine, luteolin, ergometrinine, phytosterols, 2″-O-glycosylisovitexin, arundoin, apigenin, tricin, beta-ionene, 2-coumarinate, triglochinin, ferulic acid, phenyl acetaldehyde, syringic acid, vanillic acid, l-ascorbic acid, phytol, palmitic acid, docosanoic acid, hexadecanal, tritriacontane, furfuryl alcohol, furfural, etc., which are shown to exhibit therapeutic benefits [56].

Lead compound extraction is one of the important steps in isolating the desired bioactive compounds from natural sources [57]. Unfortunately, the quantity of lead compounds produced by plants and microorganisms is very minimal [58]. Thus, we need an ideal extraction technique for separating these traces of lead compounds from the natural resources. The selection of the ideal solvent, solvent concentration, temperature, and time, as well as a suitable extraction technique, might increase the quantity as well as the quality of the bioactive compounds obtained from an endophytic fungus [12]. In this study, fungal secondary metabolites (bioactive compounds) were successfully extracted through an ultrasound-assisted extraction method using 80% ethyl acetate as the extraction solvent. This method has found extended applications in recent years, owing to its enormous benefits, including reduced energy consumption, a shorter extraction time, less active component degradation, and a higher extraction yield compared to traditional extraction methods [34].

The extract induced cytotoxicity in MCF-7 cells. The IC_50_ value against MCF-7 cells was 16.25 µg mL^−1^. This is similar to the observations made in the endophyte *Terminalia catappa,* wherein the IC_50_ against human cervical cancer HeLa cells was estimated to be 33.35 µg/mL [59]. Further analysis showed that the *A. flavus* extract induces apoptosis in the breast cancer cell line. Apoptosis is a phenomenon of programmed cell death and is the preferred type of tumor cytotoxicity in the treatment of cancer [60]. Therefore, quantitative and qualitative apoptosis assays were performed, including AO/EtBr double staining, flow cytometric analysis with Annexin V, PI, and FITC staining, along with DAPI staining assay and finally assays for changes in the mitochondrial membrane potential and measurement of ROS generation to assess and analyze apoptosis in them.

Mitochondria play a significant role in cellular functions, including the production of energy (ATP), the maintenance of Ca^2+^ ions, cell signaling, cell cycle progression, and finally cell death, as the loss of mitochondrial membrane potential and Ca^2+^ release are among the intrinsic signals triggering apoptosis. The mitochondrial membrane potential is generated by protons pumped into the inner membrane space of the mitochondria, an essential process in generating ATP. Mitochondrial membrane depolarization can be caused by an excessive generation of ROS, high intracellular calcium concentrations, or oxidative stress of the endoplasmic reticulum [61]. In this study, the IC_50_ concentration of the *A. flavus* extract triggered mitochondrial membrane potential break down. This may lead to respiratory chain uncoupling and excessive ROS production, followed by oxidative stress with oxidative damages to essential biomolecules leading to cell cycle arrest, and, finally, induced apoptotic cancer cell death [62]. It has been previously shown that *Aspergillus* extracts can lead to oxidative stress [63].

The observed cytotoxicity can be the result of the activity of the compounds present in the *A. flavus* extract. Thus, to determine precisely which components of the *A. flavus* extract are involved, a GC-MS analysis has been performed. The GC-MS spectral data demonstrated the presence of bioactive compounds that might be responsible for the anticancer activities. The obtained GC-MS spectra show the presence of nine phytochemicals, and seven of them were considered as bioactive compounds. The cytotoxic activity of one of these bioactive compounds, i.e., 1H-isoindol-1,3 (2H)-dione, against the HeLa, C6, and A549 cancer cell lines has already been established [64]. Heptadecanoic acid inhibited cell proliferation in PC9 non-small cell lung cancer cells [65]. Similarly, tritetracontane has also already been proven to have anticancer efficacies [66].

Computational drug discovery tools are helpful in identifying highly active molecules, narrowing down the biological and synthetic research needs [67]. Moreover, these tools assist in predicting the pharmacokinetic and pharmacodynamic properties of the molecules, which are subsequently confirmed by in vitro and in vivo studies [68]. In addition to that, they provide information about how these molecules bind with, interact with, donate electrons to/accept electrons from, and up or down regulate the protein/enzyme activity. This may aid researchers in developing treatment alternatives for specific diseases. Surprisingly, some of the compounds identified in the extract exhibited structural features that showed structural properties that predisposed them to possible interaction with the Bcl-2 receptor, which is overexpressed on cells of the breast cancer subtype called Bcl-2-enriched breast cancer [69]. Thus, we performed a molecular docking analysis of the bioactive compounds, and identified the one among them, i.e., 3α, 5α-cyclo-ergosta-7,9(11),22t-triene-6β-ol, which showed the highest binding affinity (−9.5 Kcal/mol) against the overexpressed anti-apoptotic protein receptor Bcl-2 of breast cancer. The binding affinity score was close to that of the standard drug Venetoclax (−10.90 Kcal/mol), which targets Bcl-2 and some other receptor kinases and is clinically used in breast cancer treatment for that purpose. Recent evidence suggests that Bcl-2 can directly influence apoptosis by translocating to the mitochondria to inhibit cytochrome c release [70]. Altogether, the compounds identified in the endophytic fungal extract have a vast range of possible anticancer mechanisms against breast tumors. Not only can they hit the universal cellular energy metabolism system by uncoupling the membranes of the mitochondria and subsequently generating ROS, but they are also potentially effective against the Bcl-2-enriched breast cancer subtype.

## 5. Conclusions

To sum up, *A. flavus* is an endophytic fungus isolated from the roots of *C. dactylon,* and its ethyl acetate extract showed a potent anticancer activity against breast cancer (MCF-7) cells. The profiling of bioactive compounds from the ethyl acetate extract of endophytic fungus was detected through GC-MS analysis, and nine compounds were identified. Among them, two phytocompounds, namely, 3α, 5α-cyclo-ergosta-7,9(11),22t-triene-6β-ol, and 2,4,7-trinitrofluorenone, exhibited the highest binding affinities, −9.50 and −9.20 Kcal/mol, respectively, to the anti-apoptotic protein (Bcl-2) receptor. Molecular dynamic simulation studies confirmed that the stability of the intermolecular interactions of the ligands−receptor binding complexes of the *A. flavus* extract exerted cytotoxic effects, significantly reducing the viability of MCF-7 cells, with an IC_50_ of 16.25 µg/mL. The extract-induced apoptosis in the MCF-7 cells was associated with a high ROS generation, nuclear material damage, and the dissipation of mitochondrial transmembrane potential. To conclude, *A. flavus,* an endophytic fungus isolated from the roots of *C. dactylon*, has the potential to be a source of novel therapeutic drugs. Additional studies are required to purify the compounds, elucidate the mechanism of action, and study its safety, so that these compounds can be developed as future drugs for cancer therapies.

## Figures and Tables

**Table 1:** Flow cytometry analysis of annexin V/PI apoptosis assay performed on MCF-7 cells treated with *A. flavus* extract.

MCF-7 Cells	Q4 Live (%)	Q3 Early Apoptosis (%)	Q2 Late Apoptosis (%)	Q1 Dead (%)
Control	90.6	0.89	5.52	2.94
EF extract	47.0	14.0	38.9	0.21

**Table 2:** Phytochemicals found in *A. flavus* extract using GC-MS.

S. No	Retention Time	% Area of Peak	Phytochemical Compounds	Molecular Formula	Molecular Weight (in g/mol)	Structure
1	23.693	0.25	2,4,7-trinitrofluorenone	C_13_H_5_N_3_O_7_	315.19	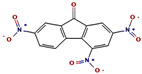
2	25.580	0.80	3,6-Bis (N-formamido) carbazole	C_14_H_11_N_3_O_2_	253.26	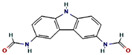
3	24.699	0.86	1H-thiopyrano[3,4-c] pyridine-5-carbonitrile	C_15_H_14_N_2_O_2_S	286.4	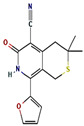
4	28.734	6.00	1H-isoindole-1,3(2H)-dione	C_13_H_10_N_2_O_4_	258.23	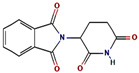
5	18.911	0.30	Heptadecanoic acid	C_17_H_34_O_2_	270.5	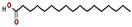
6	18.911	0.30	Methyl 2,8-dimethyltridecanoate	C_16_H_32_O_2_	256.42	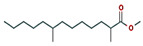
7	26.042	1.15	3α,5α-cyclo-ergosta-7,9(11),22t-triene-6β-ol	C_28_H_42_O	394.6	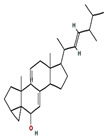
8	20.757	0.33	2,3,4-trimethyllevoglucosan	C_9_H_16_O_5_	204.22	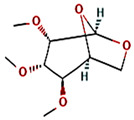
9	14.734	0.21	Tritetracontane	C_43_H_88_	605.2	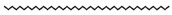

**Table 3:** Bioactive phytochemicals from *A. flavus* extract and standard drug with their binding affinity against Bcl-2 receptor (PDB id: 6O0K).

S. No	CID	Compound	Binding Affinity (Kcal/mol)
1	8521	2,4,7-trinitrofluorenone	−9.2
2	620086	3,6-Bis (N-formamido) carbazole	−6.2
3	6809	1H-isoindole-1,3(2H)-dione	−6.1
4	658451	1H-thiopyrano [3,4-c] pyridine-5-carbonitrile	−6.2
5	10465	Heptadecanoic acid	−5.4
6	560473	Methyl 2,8-dimethyltridecanoate	−5.5
7	5363271	3α, 5α-cyclo-ergosta-7,9(11),22t-triene-6β-ol	−9.5
8	91699158	2,3,4-trimethyllevoglucosan	−4.5
9	522398	Tritetracontane	−5.3
Standard drug			
10	49846579	Venetoclax	−10.9

## Data Availability

Any data or material that support the findings of this study can be made available by the corresponding author upon request.

## Supplementary Materials

The following supporting information can be downloaded at: https://www.mdpi.com/article/10.3390/molecules27248814/s1, Table S1: MTT assay; Table S2: ROS generation assay.

## References

[1] Wild C.P., Wild C.P., Stewart B.W. (2014). World Cancer Report 2014.

[2] Schiffman J.D., Breen M. (2015). Comparative oncology: What dogs and other species can teach us about humans with cancer. Philos. Trans. R. Soc. B Biol. Sci..

[3] Eccles S.A., Aboagye E.O., Ali S., Anderson A.S., Armes J., Berditchevski F., Blaydes J.P., Brennan K., Brown N.J., Bryant H.E. (2013). Critical research gaps and translational priorities for the successful prevention and treatment of breast cancer. Breast Cancer Res..

[4] McKinney S.M., Sieniek M., Godbole V., Godwin J., Antropova N., Ashrafian H., Shetty S. (2020). International evaluation of an AI system for breast cancer screening. Nature.

[5] Feng Y., Spezia M., Huang S., Yuan C., Zeng Z., Zhang L., Ji X., Liu W., Huang B., Luo W. (2018). Breast cancer development and progression: Risk factors, cancer stem cells, signaling pathways, genomics, and molecular pathogenesis. Genes Dis..

[6] Williams M.M., Cook R.S. (2015). Bcl-2 family proteins in breast development and cancer: Could Mcl-1 targeting overcome therapeutic resistance. Oncotarget.

[7] Chowdhury A., Kunjiappan S., Panneerselvam T., Somasundaram B., Bhattacharjee C. (2017). Nanotechnology and nanocarrier-based approaches on treatment of degenerative diseases. Int. Nano Lett..

[8] Runowicz C.D., Leach C.R., Henry N.L., Henry K.S., Mackey H.T., Cowens-Alvarado R.L., Cannady R.S., Pratt-Chapman M.L., Edge S.B., Jacobs L.A. (2016). American cancer society/American society of clinical oncology breast cancer survivorship care guideline. CA A Cancer J. Clin..

[9] Wang Y., Wang X., Zhao H., Liang B., Du Q. (2012). Clusterin confers resistance to TNF-alpha-induced apoptosis in breast cancer cells through NF-kappaB activation and Bcl-2 overexpression. J. Chemother..

[10] Clark A.M. (1996). Natural products as a resource for new drugs. Pharm. Res..

[11] Lam K.S. (2007). New aspects of natural products in drug discovery. Trends Microbiol..

[12] Selvaraj K., Theivendren P., Pavadai P., Ravishankar V., Palanisamy P., Gopal M., Dharmalingam S.R., Sankaranarayanan M. (2022). Impact of Physicochemical Parameters on Effective Extraction of Bioactive Compounds from Natural Sources: An Overview. Curr. Bioact. Compd..

[13] Antonelli A., Smith R.J., Fry C., Simmonds M.S.J., Kersey P.J., Pritchard H.W., Ainsworth A.M. (2020). State of the World’s Plants and Fungi.

[14] Holliday J., Cleaver M.P. (2008). Medicinal value of the caterpillar fungi species of the genus *Cordyceps* (Fr.) Link (Ascomycetes). A review. Int. J. Med. Mushrooms.

[15] Rappé M.S., Giovannoni S.J. (2003). The uncultured microbial majority. Annu. Rev. Microbiol..

[16] Cole R.J., Schweikert M.A., Jarvis B.B. (2003). Handbook of Secondary Fungal Metabolites.

[17] Dewick P.M. (2002). Medicinal Natural Products: A Biosynthetic Approach.

[18] Turner W.B. (1971). Fungal Metabolites.

[19] Kharwar R.N., Mishra A., Gond S.K., Stierle A., Stierle D. (2011). Anticancer compounds derived from fungal endophytes: Their importance and future challenges. Nat. Prod. Rep..

[20] Zhan J., Burns A.M., Liu M.X., Faeth S.H., Gunatilaka A.L. (2007). Search for cell motility and angiogenesis inhibitors with potential anticancer activity: Beauvericin and other constituents of two endophytic strains of Fusarium oxysporum. J. Nat. Prod..

[21] Yu H., Zhang L., Li L., Zheng C., Guo L., Li W., Sun P., Qin L. (2010). Recent developments and future prospects of antimicrobial metabolites produced by endophytes. Microbiol. Res..

[22] Khan A.L., Shahzad R., Al-Harrasi A., Lee I.J. (2017). Endophytic microbes: A resource for producing extracellular enzymes. Endophytes: Crop Productivity and Protection.

[23] Ratnaweera P.B., de Silva E.D., Williams D.E., Andersen R.J. (2015). Antimicrobial activities of endophytic fungi obtained from the arid zone invasive plant *Opuntia dillenii* and the isolation of equisetin, from endophytic *Fusarium* sp. BMC Complement. Altern. Med..

[24] Li C., Wang F., Wu X., Cao S. (2020). A new 24-homo-30-nor-cycloartane triterpenoid from a Hawaiian endophytic fungal strain. Tetrahedron Lett..

[25] Kaliyaperumal A., Kumarakurubaran S., Saradha D.M. (2013). *Cynodon dactylon* (L.) Pers.: An updated review of its phytochemistry and pharmacology. J. Med. Plants Res..

[26] Kowsalya R., Kaliaperumal J., Vaishnavi M., Namasivayam E. (2015). Anticancer activity of *Cynodon dactylon* L. root extract against diethyl nitrosamine induced hepatic carcinoma. South Asian J. Cancer.

[27] Kleensang A., Vantangoli M.M., Odwin-DaCosta S., Andersen M.E., Boekelheide K., Bouhifd M., Fornace A.J., Li H.-H., Livi C.B., Madnick S. (2016). Genetic variability in a frozen batch of MCF-7 cells invisible in routine authentication affecting cell function. Sci. Rep..

[28] Tapfuma K.I., Uche-Okereafor N., Sebola T.E., Hussan R., Mekuto L., Makatini M.M., Green E., Mavumengwana V. (2019). Cytotoxic activity of crude extracts from Datura stramonium’s fungal endophytes against A549 lung carcinoma and UMG87 glioblastoma cell lines and LC-QTOF-MS/MS based metabolite profiling. BMC Complement. Altern. Med..

[29] Sette L.D., Passarini M.R.Z., Delarmelina C., Salati F., Duarte M.C.T. (2006). Molecular characterization and antimicrobial activity of endophytic fungi from coffee plants. World J. Microbiol. Biotechnol..

[30] Kunjiappan S., Theivendran P., Baskararaj S., Sankaranarayanan B., Palanisamy P., Saravanan G., Arunachalam S., Sankaranarayanan M., Natarajan J., Somasundaram B. (2019). Modeling a pH-sensitive Zein-co-acrylic acid hybrid hydrogels loaded 5-fluorouracil and rutin for enhanced anticancer efficacy by oral delivery. 3 Biotech.

[31] Kalimuthu A.K., Parasuraman P., Sivakumar P., Murugesan S., Arunachalam S., Pandian S.R.K., Ravishankar V., Ammunje D.N., Sampath M., Panneerselvam T. (2022). In silico, in vitro screening of antioxidant and anticancer potentials of bioactive secondary metabolites from an endophytic fungus (*Curvularia* sp.) from *Phyllanthus niruri* L. Environ. Sci. Pollut. Res..

[32] Mohan U.P., Sriram B., Panneerselvam T., Devaraj S., MubarakAli D., Parasuraman P., Palanisamy P., Premanand A., Arunachalam S., Kunjiappan S. (2020). Utilization of plant-derived Myricetin molecule coupled with ultrasound for the synthesis of gold nanoparticles against breast cancer. Naunyn-Schmiedeberg’s Arch. Pharmacol..

[33] Kunjiappan S., Sankaranarayanan M., Kumar B.K., Pavadai P., Babkiewicz E., Maszczyk P., Glodkowska-Mrowka E., Arunachalam S., Pandian S.R.K., Ravishankar V. (2020). Capsaicin-loaded solid lipid nanoparticles: Design, biodistribution, in silico modeling and in vitro cytotoxicity evaluation. Nanotechnology.

[34] Kunjiappan S., Panneerselvam T., Govindaraj S., Kannan S., Parasuraman P., Arunachalam S., Sankaranarayanan M., Baskararaj S., Palanisamy P., Ammunje D.N. (2020). Optimization and analysis of ultrasound-assisted extraction of bioactive polyphenols from Garcinia indica using RSM and ANFIS modeling and its anticancer activity. J. Iran. Chem. Soc..

[35] Release S. (2017). 3: Desmond molecular dynamics system, DE Shaw research, New York, NY, 2017. Maestro-Desmond Interoperability Tools.

[36] Jorgensen W.L., Maxwell D.S., Tirado-Rives J. (1996). Development and testing of the OPLS all-atom force field on conformational energetics and properties of organic liquids. J. Am. Chem. Soc..

[37] Jorgensen W.L., Chandrasekhar J., Madura J.D., Impey R.W., Klein M.L. (1983). Comparison of simple potential functions for simulating liquid water. J. Chem. Phys..

[38] Basha S.H., Bethapudi P., Majji Rambabu F. (2014). Anti-angiogenesis property by Quercetin compound targeting VEGFR2 elucidated in a computational approach. Eur. J. Biotechnol. Biosci..

[39] Nosé S. (1984). A unified formulation of the constant temperature molecular dynamics methods. J. Chem. Phys..

[40] Gunjan M., Naing T.W., Saini R.S., Ahmad A., Naidu J.R., Kumar I. (2015). Marketing trends & future prospects of herbal medicine in the treatment of various disease. World J. Pharm. Res..

[41] Ijäs H., Shen B., Heuer-Jungemann A., Keller A., Kostiainen M.A., Liedl T., Ihalainen J.A., Linko V. (2021). Unraveling the interaction between doxorubicin and DNA origami nanostructures for customizable chemotherapeutic drug release. Nucleic Acids Res..

[42] Sadananda T., Govindappa M., Ramachandra Y., Chandrappa C., Umashankar T. (2016). In Vitro Apoptotic Activity of Endophytic Fungal Lectin Isolated from Endophyte, Aspergillus flavus of Viscum album on Human Breast Adenocarcinoma Cell Line (MCF-7). Metabolomics.

[43] Pereira S., Castro P. (2014). Diversity and characterization of culturable bacterial endophytes from Zea mays and their potential as plant growth-promoting agents in metal-degraded soils. Environ. Sci. Pollut. Res..

[44] Siddiqui M., Rajkumar S.V. (2012). The high cost of cancer drugs and what we can do about it. Mayo Clinic Proceedings.

[45] Pal S.K., Shukla Y. (2003). Herbal medicine: Current status and the future. Asian Pac. J. Cancer Prev..

[46] Dakh K.S., Patekar R.R., Choudhary H.B., Momin A.Z., Undale V.R., Mahadik P., Desai S., Asalkar M., Shaikh H. (2022). Herbal approach for tuberculosis management: A systematic review. World J. Adv. Res. Rev..

[47] Tabish S.A. (2008). Complementary and alternative healthcare: Is it evidence-based?. Int. J. Health Sci..

[48] Amador M.L., Jimeno J., Paz-Ares L., Cortes-Funes H., Hidalgo M. (2003). Progress in the development and acquisition of anticancer agents from marine sources. Ann. Oncol..

[49] Rayan A., Raiyn J., Falah M. (2017). Nature is the best source of anticancer drugs: Indexing natural products for their anticancer bioactivity. PLoS ONE.

[50] Das K., Tiwari R.K.S., Shrivastava D.K. (2010). Techniques for evaluation of medicinal plant products as antimicrobial agent: Current methods and future trends. J. Med. Plants Res..

[51] Partida-Martínez L.P., Heil M. (2011). The microbe-free plant: Fact or artifact?. Front. Plant Sci..

[52] Chandra S. (2012). Endophytic fungi: Novel sources of anticancer lead molecules. Appl. Microbiol. Biotechnol..

[53] Sopalun K., Laosripaiboon W., Wachirachaikarn A., Iamtham S. (2021). Biological potential and chemical composition of bioactive compounds from endophytic fungi associated with thai mangrove plants. South Afr. J. Bot..

[54] Firáková S., Šturdíková M., Múčková M. (2007). Bioactive secondary metabolites produced by microorganisms associated with plants. Biologia.

[55] Nagori B.P., Solanki R. (2011). *Cynodon dactylon* (L.) Pers.: A valuable medicinal plant. Res. J. Med. Plant.

[56] Selvaraj K., Chowdhury R., Bhattacharjee C. (2014). Optimization of the solvent extraction of bioactive polyphenolic compounds from aquatic fern *Azolla microphylla* using response surface methodology. Int. Food Res. J..

[57] Ganesan V., Gurumani V., Kunjiappan S., Panneerselvam T., Somasundaram B., Kannan S., Chowdhury A., Saravanan G., Bhattacharjee C. (2018). Optimization and analysis of microwave-assisted extraction of bioactive compounds from *Mimosa pudica* L. using RSM & ANFIS modeling. J. Food Meas. Charact..

[58] Cragg G.M., Newman D.J. (2013). Natural products: A continuing source of novel drug leads. Biochim. Biophys. Acta (BBA)-Gen. Subj..

[59] Majoumouo M.S., Tincho M.B., Toghueo R.M.K., Morris T., Hiss D.C., Boyom F.F., Mandal C. (2020). Cytotoxicity potential of endophytic fungi extracts from Terminalia catappa against human cervical cancer cells. J. Toxicol..

[60] Elmore S. (2007). Apoptosis: A review of programmed cell death. Toxicol. Pathol..

[61] Taggart L.E., McMahon S.J., Currell F.J., Prise K.M., Butterworth K.T. (2014). The role of mitochondrial function in gold nanoparticle mediated radiosensitisation. Cancer Nanotechnol..

[62] Redza-Dutordoir M., Averill-Bates D.A. (2016). Activation of apoptosis signalling pathways by reactive oxygen species. Biochim. Biophys. Acta (BBA)-Mol. Cell Res..

[63] Tan A., Yaglioglu A.S., Kishali N.H., Sahin E., Kara Y. (2020). Evaluation of cytotoxic potentials of some isoindole-1, 3-dione derivatives on HeLa, C6 and A549 cancer cell lines. Med. Chem..

[64] Xu C., Wu P., Gao J., Zhang L., Ma T., Ma B., Yang S., Shao G., Yu Y., Huang X. (2019). Heptadecanoic acid inhibits cell proliferation in PC9 nonsmallcell lung cancer cells with acquired gefitinib resistance. Oncol. Rep..

[65] Rhetso T., Shubharani R., Roopa M.S., Sivaram V. (2020). Chemical constituents, antioxidant, and antimicrobial activity of Allium chinense G. Don. Future J. Pharm. Sci..

[66] Ban F., Dalal K., Li H., LeBlanc E., Rennie P.S., Cherkasov A. (2017). Best practices of computer-aided drug discovery: Lessons learned from the development of a preclinical candidate for prostate cancer with a new mechanism of action. J. Chem. Inf. Model..

[67] Macalino S.J.Y., Gosu V., Hong S., Choi S. (2015). Role of computer-aided drug design in modern drug discovery. Arch. Pharm. Res..

[68] Baig M.H., Ahmad K., Rabbani G., Danishuddin M., Choi I. (2018). Computer aided drug design and its application to the development of potential drugs for neurodegenerative disorders. Curr. Neuropharmacol..

[69] Kønig S.M., Rissler V., Terkelsen T., Lambrughi M., Papaleo E. (2019). Alterations of the interactome of Bcl-2 proteins in breast cancer at the transcriptional, mutational and structural level. PLoS Comput. Biol..

[70] Kuwana T., Newmeyer D.D. (2003). Bcl-2-family proteins and the role of mitochondria in apoptosis. Curr. Opin. Cell Biol..

