# Supplementary material for: Cytotoxic Potential of Bioactive Compounds from Aspergillus flavus, an Endophytic Fungus Isolated from Cynodon dactylon, against Breast Cancer: Experimental and Computational Approach
Source: Molecules. 2022 Dec 12;27(24):8814. doi: 10.3390/molecules27248814 (PMC9784115; doi:10.3390/molecules27248814)
Supplement: Supplementary file 1 [file molecules-27-08814-s001.zip › molecules-2082731-supplementary.pdf]

Supplementary Data for

Cytotoxic and cytostatic potential of bioactive secondary metabolites from *Aspergillus flavus*, an endophytic fungus isolated from *Cynodon dactylon* against breast cancer*: in vitro* and *in silico* studies

Arjun Kumar Kalimuthu[1], Pavadai Parasuraman[2], Theivendren Panneerselvam[3], Ewa Babkiewicz[4], Joanna Pijanowska[4], Piotr Mrowka[5,6], Gopalan Rajagopal[7], Venkataraman Deepak[1,8], Krishnan Sundar[1*], Piotr Maszczyk[4]*, Selvaraj Kunjiappan[1*]

[1]Department of Biotechnology, Kalasalingam Academy of Research and Education, Krishnankoil-626126, India.

[2]Department of Pharmaceutical Chemistry, Faculty of Pharmacy, M.S. Ramaiah University of Applied Sciences, Bengaluru-560054, India.

[3]Department of Pharmaceutical Chemistry, Swamy Vivekanandha College of Pharmacy, Elayampalayam-637205, India.

[4]Department of Hydrobiology, Faculty of Biology, University of Warsaw at Biology and Chemistry Research Center, 02-089 Warsaw, Poland.

[5]Department of Biophysics, Physiology and Pathophysiology, Medical University of Warsaw, 5 Chalubinskiego Street, 02-004 Warsaw, Poland.

[6]Department of Experimental Hematology, Institute of Hematology and Transfusion Medicine, 5 Chocimska Street, 00-791 Warsaw, Poland.

[7]Postgraduate and Research Department of Zoology, Ayya Nadar Janaki Ammal College, Sivakasi-626123, India.

[8]Maternal and Fetal Health Research Centre, 5th Floor St. Mary's Hospital, University of Manchester, Oxford Road Manchester, M13 9WL, United Kingdom.

***Correspondence address:**

Krishnan Sundar: sundarkr@klu.ac.in; Ph: +91 9486953248

Piotr Maszczyk: fizbanek@wp.pl; Ph: +48 22 55 26568

Selvaraj Kunjiappan; selvapharmabio@gmail.com; Ph: +91 9994972108

This MS word file includes:

Supplementary Tables S1 to S2.

Table S1: MTT assay

Conc	control	3.125	6.25	12.5	25	50	100	Dox 0.25 µM
	100	85.12	64.84	53.19	47.45	38.24	28.09	45
		86.49	64.04	52.66	47.45	38.24	26.49	43.67
		87.29	60.85	53.99	48.24	39.31	26.22	43.14

Group Name	N	Missing	Mean	Std Dev	SEM
			100	0	0
Col 1	3	0	86.3	1.097	0.634
Col 2	3	0	63.243	2.111	1.219
Col 3	3	0	53.28	0.67	0.387
Col 4	3	0	47.713	0.456	0.263
Col 5	3	0	38.597	0.618	0.357
Col 6	3	0	26.933	1.011	0.584
Col 7	3	0	43.937	0.958	0.553
Source of Variation	DF	SS	MS	F	P
Between Groups	6	6580.917	1096.82	888.816	<0.001
Residual	14	17.276	1.234		
Total	20	6598.193			

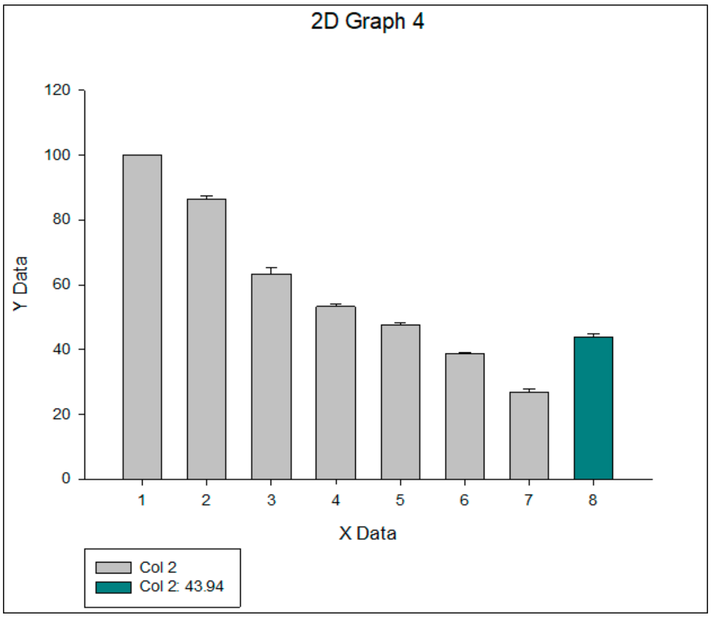

Table S2: ROS generation assay

			ctrl	24	48
			21.34	55.63	72.87
			20.56	54.34	73.38
			22.71	56.58	71.41
Group Name	N	Missing	Mean	Std Dev	SEM
Col 2	3	0	21.537	1.088	0.628
Col 3	3	0	55.517	1.124	0.649
Col 4	3	0	72.553	1.022	0.59
Source of Variation	DF	SS	MS	F	P
Between Groups	2	4047.589	2023.794	1737.61	<0.001
Residual	6	6.988	1.165		
Total	8	4054.577			

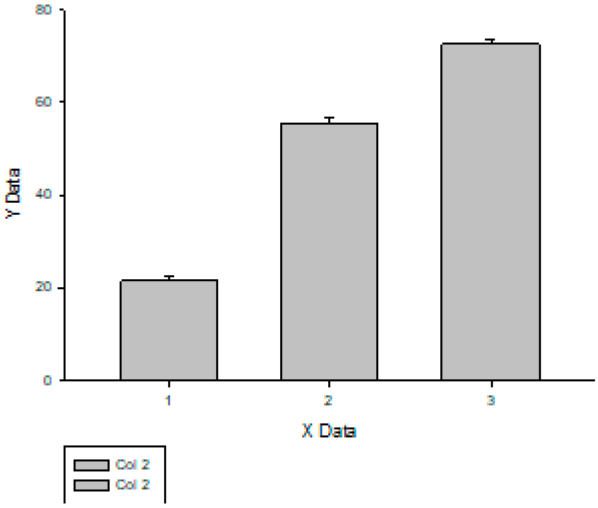

80
60
40
20
0
Y Data
1
2
3
X Data
Col 2
Col 2
